# Knowledge, Attitude and Smoking Patterns Among Pregnant Women: A Jordanian Perspective

**DOI:** 10.5334/aogh.3279

**Published:** 2021-04-06

**Authors:** Jehan Hamadneh, Shereen Hamadneh, Zouhair Amarin, Soha Al-Beitawi

**Affiliations:** 1 Department of Obstetrics and Gynecology, Faculty of Medicine, Jordan University of Science and Technology, Irbid, Jordan; 2 Department of Maternal and Child Health, Princess Salma Faculty of Nursing, Al al-Bayt University, Mafraq, Jordan; 3 Department of Clinical Sciences, Faculty of Medicine, Yarmouk University, Irbid, Jordan

## Abstract

**Background:**

Smoking during pregnancy is one of the main modifiable factors associated with perinatal morbidity and mortality and maternal complications. Literature is scant regarding smoking habits of pregnant women in Jordan.

**Objectives:**

To investigate smoking patterns and attitudes of Jordanian pregnant women towards smoking.

**Methods:**

A cross-sectional survey of patterns and attitudes towards smoking among 436 mothers attending healthcare facilities in the Governorate of Irbid, Jordan, between August and September 2019.

**Results:**

Out of 436 pregnant women in the Governorate of Irbid, Jordan, 13 (2.9%) quit smoking once pregnancy was conformed, and 77 (17.6%) continued to smoke.

Pregnant non-smokers believed that hookah and electronic cigarettes are as bad to health as cigarettes, while smokers believed that hookah and electronic cigarettes are less hazardous than cigarettes (5.19% versus 21.99%, p = 0.001, and 6.49% versus 19.37%, p = 0.009, respectively).

Non-smokers were significantly more aware regarding the hazards of smoking on perinatal outcomes, such as abortion (31.94% versus 10.39%, p = 0.001), placental abruption (31.94% versus 10.39%, p = 0.001), intrauterine growth restriction and fetal malformations (36.65% versus 14.29%, p = 0.007), fetal death (30.89% versus 6.49, p < 0.001), neonatal pulmonary diseases (44.50% versus 24.68%, p = 0.024), neonatal asthma (47.12% versus 28.57%, p = 0.038), and ear diseases (42.41% versus 20.8%, p = 0.012).

**Conclusion:**

There is a high prevalence of smokers among pregnant women in Jordan. The level of awareness regarding long-term consequences of tobacco use remains low. Educational programs should include information about the hazards of all forms of smoking. Pregnancy provides a good opportunity for promoting smoking cessation.

## Introduction

Smoking during pregnancy is one of the main preventable factors associated with increased risk for maternal and fetal morbidity and mortality [1,2].

In the United States, despite a decline in the overall prevalence of cigarette smoking [3], smoking rates before and during pregnancy have not changed much [4]. About 20% of women aged between 18 and 44 years are smokers [5], and three fourths of them smoked during pregnancy [6].

In most developed countries, the prevalence of smoking during pregnancy has been changing. In the United Kingdom, the rates of cigarette smoking at the time of delivery have declined from 15% in 2007 to 12% in 2014 [7]. Australia [8], Canada [9] and the USA have comparable rates of smoking during pregnancy of about 10% [6], while in Ireland, Uruguay and Bulgaria, the rates are between 29% and 38%. This is significantly higher than the current global average rate of 1.7% [10].

Recent data suggest that the main predictors for smoking during pregnancy are younger maternal age [11,12,13], low socioeconomic factors [14,15,16], lower levels of education [12], shift work and being unemployed [17].

Generally, there is a dearth of studies that cover the level of knowledge regarding the dangers of smoking during pregnancy. Reported awareness of the adverse effects of smoking is significantly less in smokers than non-smokers [13,18,19]. Studies show a better understanding of smoking-related risks, such as miscarriage, low birth weight, neonatal illness and childhood behavioral problems, in non-smoking pregnant women compared to smokers [20,21]. These factors would be similar to those that define smoking among non-pregnant women in general and the prevalence of smoking in women who become pregnant. In addition, pregnancy may influence smoking behavior in some way.

One of the healthcare system’s key objectives is to decrease smoking during pregnancy to a target rate of 1–2% [22]. To develop targeted interventions, more knowledge is needed about the characteristics of women who quit and those who continue to smoke during pregnancy. While there are studies, albeit not recent, on the prevalence of smoking in the general adult population, we do not have a perspective about smoking behavior among women of reproductive age or those that are pregnant in Jordan.

This study aimed to investigate the smoking patterns and attitudes of Jordanian pregnant women and their knowledge about maternal and fetal risk factors associated with tobacco use during pregnancy.

## Methods

This study was conducted in hospitals that provide obstetric care in the Governorate of Irbid. It consisted of three stages:

1. Evaluation of smoking prevalence among pregnant women.
2. Assessment of the knowledge and attitudes of pregnant women regarding the health risks of smoking.
3. Identifying factors affecting the rate of smoking among pregnant women.

This is a cross-sectional survey using a semi-structured questionnaire (with both open-ended and closed-ended questions) to describe smoking behaviors, knowledge, patterns, and attitudes of smoking among all mothers attending main hospitals and healthcare facilities in the Governorate of Irbid (King Abdullah University Hospital, Princess Badea Teaching Hospital, Qawasmi Obstetric Hospital, and Specialty Hospital) between August and September 2019.

Smoking status was defined for each product (cigarettes, hookah, e- cigarettes) based on use in the last two months, that included daily, non-daily or frequency of tobacco use per week.

Quitting smoking, for the purpose of this study, was defined as the process of stopping tobacco use since the diagnosis of current pregnancy.

Inclusion criteria were being pregnant, consenting to participate in the research and completing at least 75% of the questionnaire.

Sample size was calculated according to the Hajian-Tilaki K. (2011):

n = \frac{{{{({z_{ \propto /2}})}^2}p\,(1-p)}}{{{\Delta ^2}}}

Where n = sample size, z = z-score (the number of standard deviations a given proportion is away from the mean) with a coefficient for confidence interval of 95% (was 1.96), p = the expected proportion of smokers among pregnant women, D = maximal marginal error.

No studies on the prevalence of smoking during pregnancy are available for Jordan. Based on studies across international populations in the published literature, the maximum prevalence of smoking among pregnant women was 38% [10]. The maximum marginal error was equal to 5%.

The calculated sample size was 334.

The Institutional Research Board of Jordan University of Science and Technology approved the study. All participants who agreed to participate in this study were interviewed using an adapted questionnaire that was developed for Middle Eastern women [23]. The questionnaire included Yes/No, select from a list and short answer questions.

In this face-to-face survey, the measured variables included socio-demographic characteristics, knowledge, attitudes and smoking patterns with questions on the impact of smoking on perinatal outcome.

Statistical analysis was performed using SPSS 25 (IBM Company, New York, USA). The character of data distribution was evaluated with W-criterion of Shapiro-Wilk. Mann-Whitney U-test was used for comparison of two independent groups about the equality of middle rank. Analysis of contingency tables (χ2) was used to assess the differences in relative values. Fisher exact p was applied at frequencies less than 5. Comparison of the relative frequencies in the two groups was carried out by comparing the 95% CI relative frequencies. The odds ratio (OR) with 95% confidence interval (CI) was used to estimate the odds of smoking correlated with social-economic and clinical factors. The threshold for statistical significance was set at p < 0.05.

## Results

A total of 436 met the inclusion criteria: 268 pregnant women completed the questionnaire, 191 were non-smokers, 13 (2.9%) quit smoking once pregnancy was confirmed, 77 (17.6%) were smokers during the index pregnancy.

### Brief characteristic of the studied group

The average maternal age was 31 years. Jordanians represented 65%, and 44% had no high education (bachelor degree or higher), 56.7% were housewives. 92.16% had working husbands. Fifty-six percent felt that their family income was insufficient, 58% were nulliparous and 64% were not regular attendees of antenatal clinics (less than three visits all through the antenatal period).

### Prevalence of smoking during pregnancy

The current study showed that the prevalence of smoking among pregnant women in Irbid governorate is 17.6%. The type of smoking among the smokers during pregnancy is presented in ***Table 1***.

**Table 1:** Pattern of smoking among 77 pregnant women in the Governorate of Irbid.


TYPE OF SMOKING	NUMBER (%)

Cigarettes	9 (11.6)

Hookah	17 (22.0)

Electronic cigarettes	8 (10.3)

Mixed	43 (55.8)


### Knowledge among pregnant women regarding the effects of smoking

There was no statistical difference among smokers (74.03%) and non-smokers (80.10%) who believed that smoking during pregnancy affects the fetus. Less than half (42.9%) of respondents believed that electronic cigarettes produce hazardous gases, and 43.98% thought that these devices have no ill-effects on health.

Non-smokers believed hookah and electronic cigarettes have the same negative effect on health as cigarettes, in contrast to smokers: 21.99% versus 5.19% (p = 0.001) and 19.37% versus 6.49% (p = 0.009), respectively.

Non-smokers were significantly more aware of the effect of smoking cigarettes and hookah on perinatal outcomes: abortion 31.94% versus 10.39% (p = 0.001), placental abruption 31.94% versus 10.39% (p = 0.001), intrauterine growth restriction and fetal malformations 36.65% versus 14.29% (p = 0.007 for both), stillbirth 30.89% versus 6.49 (p < 0.001), neonatal pulmonary diseases 44.50% versus 24.68% (p = 0.024), neonatal asthma 47.12% versus 28.57% (p = 0.038) and auditory complications 42.41% versus 20.8% (p = 0.012).

### Association between social-economic and clinical factors and smoking during pregnancy

Our study revealed there was an association between level of education and smoking behavior. Pregnant women who smoked during pregnancy were significantly more educated than non-smokers (OR = 122.85, p < 0.0001). Maternal age, nationality, working status of husband and wife, family income, and being a regular attender of antenatal clinics were similar in two groups (***Table 2***).

**Table 2:** Association between socio-economic and smoking status of 268 pregnant women in the Governorate of Irbid.


CHARACTERISTICS	% SMOKERS (n = 77)	% NON-SMOKERS (n = 191)	OR (95% CI)	Δ^2^	P-VALUE

Mean age (years)	31.7	31.3	–	–	0.635

Jordanian Nationality	68.8	63.3	1.28 (0.73–2.24)	0.72	0.396

>12-years education	98.7	38.2	122.85 (16–902]	–	<.0001*

Working wife	40.2	44.5	0.84 (0.49–1.44)	0.40	0.527

Working husband	90.9	92.6	0.79 (0.31–2.04)	0.24	0.624

Multigravida	77.9	70.1	1.50 (0.81–2.79)	1.66	0.198

No regular antenatal care	61.0	64.9	0.85 (0.49–1.46)	0.36	0.548


* Fisher exact probability test.

## Discussion

Smoking during pregnancy is one of the main factors associated with increased perinatal and maternal morbidity [1,2]. It is strongly associated with reduced fetal measurements [24,25], low birth weight [26,27,28], wheezing and asthma in children [29].

In a study by Hamadneh and colleagues, one of the main risk factors for sudden infant death syndrome in Middle Eastern countries was smoking [30,31]. These results were confirmed by later studies [32].

Despite the many studies that have addressed smoking during pregnancy as the main risk factor of perinatal morbidity and mortality, there is a dearth of studies from Middle Eastern countries in general, and from Jordan in particular. To our knowledge, this is the first population-based study assessing the prevalence of maternal tobacco use during pregnancy in Jordan.

The study found that the prevalence of maternal tobacco use during pregnancy was significantly higher than that of the current global average rate of 1.7%, and that of the Eastern Mediterranean Region’s rate of 0.9% [10]. In contrast, it is lower than that of Italy at 23%, [33] and Poland at 34.6% [34]. On the other hand, the percentage of women who quit smoking during pregnancy was lower than the 7.6% as reported by Frandsen and colleagues in 2017 [35].

Approximately half of the pregnant smokers consumed alternative forms of tobacco, such as hookah and e-cigarettes, where 75% believed that they were less harmful and do not affect perinatal outcomes. This is comparable to other studies [36], when these alternative forms of tobacco consumption are not less harmful than traditional cigarette smoking [37]. Therefore, interventions aimed at smoking cessation should include the alternative forms of tobacco consumption.

In this study, a large proportion of pregnant smokers were not aware that smoking during pregnancy increases the risk of spontaneous abortion, intrauterine growth restriction, fetal malformation, stillbirth, neonatal asthma and auditory complications. These findings are consistent with other studies showing that pregnant smokers possess superficial knowledge of the hazards of smoking during pregnancy and that their awareness is significantly less compared to pregnant non-smokers [13,18,19,21].

The risk factors for smoking during pregnancy in the current study were not fully consistent with the findings of other studies. Our study revealed that women that are more educated were significantly more likely to smoke. The main risk factors for smoking during pregnancy were maternal age less than 25 years [11,12,13], low socioeconomic factors [14,15,16], poor level of education [12], shift work and being unemployed [17], residing in regional or remote areas, increased parity, poor attendance of antenatal clinics [38], smoking partners and a higher level of daily stress [39]. These differences could be attributable to sociocultural differences, including values, customs, taboos and trends between studied populations of pregnant women.

It is suspected that cigarette smokers are less likely to report their belief that smoking causes disease and that heavy smokers are less likely to report that belief than light smokers. This is despite abundant education where non-smokers are more likely to believe that smoking causes disease.

This effect is probably secondary to the influence of addiction on the processing of risk information regarding the use of all forms of nicotine intake. This is compounded by advertising and industry denial of disease risks. The effect of nicotine on information processing appears to be highly resistant to simply providing more education. The combination of educational input in conjunction with the provision of smoking cessation assistance would be helpful.

## Conclusion

This study revealed a high prevalence of smoking among pregnant women, and alternative forms of tobacco consumption are high, with a limited knowledge of the health hazards of all forms of smoking during pregnancy. These results call for the development and implementation of new tools to improve the promotion of smoking cessation during pregnancy.
